# The Fruits of Vivisection

**Published:** 1892-11-19

**Authors:** Lawson Tait


### The Fruits of Vivisection.

A Reply to Sir Andrew Clark, Bart.

By Lawson Tait, F.R.C.S., LL.D., &c.

Sir Andrew Clark begins by an assertion that the law of vicarious sacrifice is a law of the physical, mental, moral, and spiritual life. If the poor dogs and rabbits for whom he claims the merit of vicarious sacrifice had any knowledge of what they were doing, had votes on the question of their torture, and could escape if they wished it, I could admit that there was some clear association of the "vicarious sacrifice" with their sufferings, for Torquemada's Jews could yield their wealth and leave Spain. But Ferrier's monkeys had no choice, and could not escape.

Sir Andrew further says: "Experiments performed upon animals with due care, for a high purpose, in a reverent spirit, and with results in good transcending any suffering inflicted, are not cruel, and so on. At the beginning of the next paragraph he says it is folly now to argue the question whether experimental research has contributed to the advancement of theoretical and practical knowledge. But I submit that this is the whole question. Sir Andrew makes assertions: he must prove them. He asserts a right, and he must prove it, that right being to cause other creatures to suffer in our service. It cannot be admitted for his argument merely that it has been always and everywhere assumed and practised; and the reason against such an admission is given in Sir Andrew's own words, that "cruelty lies in the wanton infliction of suffering, and in the performance of experiments carelessly designed or barren in results of good justifying the suffering which has been inflicted." As a rough example of a large number let me take the experiments of Rutherford, Bennett, and Gamgee, which were wanton in their number (nearly seven hundred animals were sacrificed), were carelessly designed, and were not only utterly "barren in results of good justifying the enormous suffering involved," but the conclusions derived from them were absolutely false.

Sir Andrew says that it is certain that no substantial advance has been made in physiological knowledge, and none in the departments of midwifery, surgery, and medicine without the assistance of this method of research. I deny this statement altogether; but even if I admitted it, Sir Andrew has yet to overcome the difficult question, was that assistance absolutely essential for the advance? Was it not possible to dispense with it? If Sir Andrew will give categorically his examples, I shall carefully consider them and show a fallacy, if I can find one, as I feel sure I can.

He takes first of all the worn-out story of Harvey, who did not discover the circulation of the blood. If he had discovered it the result would have been in spite of his vivisectional blunders. He next says that the mortality in childbed in Vienna was at one time 16 per cent.—it was over 30 per cent. at the Krankenhaus, where alone the high mortality existed. Outside that pest-house there is no reason to believe the puerperal mortality amounted to more than 1 per cent. He says that by long and patient study, assisted by experiment upon animals, the mortality was reduced to under 1 per cent. I challenge Sir Andrew to produce his authority. Semelweiss, who discovered the cause, never made a single experiment on an animal for the purpose, at least, he gave none as his reason. He merely noticed that the women who died were nearly all attended or examined by students who came straight from the charnel houses. He made them wash their hands in a solution of chlorine, and at once the mortality fell to less than a third, but it is not one per cent. yet.

Sir Andrew's next illustration is amputation in the time of Ambrose Parè, and its results in the present day, and the differences, he says, were brought about entirely through the knowledge acquired by experiments upon living animals. But Ambrose Parè's method of ligature in amputations was retained in every day use till 1878. From 1703 till 1878 a vast array of experimenters on the blood vessels appear (I regret to say my own name will be found on the list), making confusion worse confounded by the discrepancy of their results. It was not till common sense prevailed, till plain pure silk was used for the ligature, and both ends cut short, that any real progress was made. The improvements have been due to hygiene and clinical observations.

Of the operation of ovariotomy exactly the same thing is true, and from 1809 till 1878 there is nothing but a story of failure, because men would go on experimenting on animals and would not listen to the commen-sense teaching of such men as Houston, Macdowell, Nathan Smith, and Baker Brown.

Sir Andrew further ventures on other surgical examples as instances of the benefits of vivisection: Operations on the stomach—I am inclined to grant this, as they are all failures and now almost given up. Amputating the larynx—an utterly abominable operation. Removal of one kidney, which was introduced by Simon, of Heidelberg, and Campbell, of Dundee, as the result of surgical mistakes, in both instances turned into surgical triumphs, and having as much to do with vivisection as they had to do with the Song of Solomon. Of his surgical examples, the last is the worst—the extirpation of portions of the brain and spinal cord. But a great deal of brain surgery was done before Sir Andrew was born. I can show him a list of over fifty cases of brain operations performed between 1806 and 1865. The early attempts to ocalise brain tumours were ineffective, but in the *Medical Times and Gazette* of September 8th, 1883, we have evidence that, "Now, thanks to the careful clinical observation and research and accurate pathological study of such men as Broca, Charcot, Flechsig, and others, we are beginning to see that each convolution has its special function, and no other." We have also the evidence of the famous Editor of the *British Medical Journal*, Mr. Ernest Hart, in the Journal for December 27th, 1884 (the year following that of the evidence thereby corroborated), that the results of the vivisectional experiments in the researches of Munk, Christismi, Goltz, and Lort are a "maze of conflicting statements." Mr. Horsley's celebrated successful case, which died at about the end of a month in the same year, brought out the fact that the greatest living surgeon, Professor William Macewen, of Glasgow, had for eight years been doing operations of the same kind, but only far more brilliant and far more successful, in which the splendid diagnoses and triumphant treatment were due to "a careful study of the symptoms."

Sir Andrew next asserts that we have discovered the nature and relations of infectious diseases, and learned how in some measure to prevent the development and to control the spread of fevers, cholera, anthrax and septicæmia. I cannot even guess to what he alludes in this assertion. If he will give particulars I shall look into his case, but it seems to me to carry absurdity on the face of it.

Finally, Sir Andrew says, "For your pleasure, or for your profit, or for any other object than the promotion of knowledge, you may without let or hindrance beat, starve, mutilate, or destroy as many animals as you please." But if you beat or starve an animal to the point of torture, you will be punished. What he means is that, for purposes of sport, many cruel things are done. But those who vivisect do so in the holy name of Science, and surely cannot seriously mean to support it by pleading the example of ignoble sport! If vivisection is to be defended, it is not to be done by ancient history or inaccurate statements concerning modern surgery. In the domain of the practice of medicine Sir Andrew Clark may find refuge, but in the former two matters I am at home.

